# A Silver Yarn-Incorporated Song Brocade Fabric with Enhanced Electromagnetic Shielding

**DOI:** 10.3390/ma14143779

**Published:** 2021-07-06

**Authors:** Xiuling Zhang, Zimin Jin, Lizhu Hu, Xinyi Zhou, Kai Yang, Dana Kremenakova, Jiri Militky

**Affiliations:** 1 Jiangxi Center for Modern Apparel Engineering and Technology, Jiangxi Institute of Fashion Technology, Nanchang 330201, China; xiuling.zhang@tul.cz (X.Z.); hulizhulove@163.com (L.H.); zhouxinyi01280513@163.com (X.Z.); 2 Department of Material Engineering, Faculty of Textile Engineering, Technical University of Liberec, 461 17 Liberec, Czech Republic; kai.yang@tul.cz (K.Y.); dana.kremenakova@tul.cz (D.K.); jiri.militky@tul.cz (J.M.); 3 College of Textile Science and Engineering, Zhejiang Sci-Tech University, Xiasha Education Park, Hangzhou 310018, China

**Keywords:** Song Brocade fabric, surficial pattern, electromagnetic shielding, UV protection, air permeability

## Abstract

The fabrics with electromagnetic interference (EMI) have been used in various fields. However, most studies related to the EMI fabrics focused on the improvement of the final electromagnetic shielding effectiveness (EM *SE*) by adjusting the preparation parameters while the breathability of the EMI fabrics was affected and the visible surficial patterns on the EMI fabric was limited. In this work, the two samples based on the Song Brocade structure were fabricated with surficial visible pattern ‘卐’. One was fabricated with silver-plated polyamide (Ag-PA) yarns and the silk yarns, the another with polyester (PET) yarns and the silk yarns. The weaving structure of the two samples were investigated by scanning electronic microscopy (SEM) and laser optical microscopy (LOM). The resistance against the EM radiation near field communication (NFC) and the ultraviolet (UV) light was also evaluated. Besides, the surface resistance, the air permeability and the water evaporation rate were investigated. The results revealed that the ‘卐’ appeared successfully on the surface of the two samples with stable weaving structure. The Ag-PA yarn-incorporated Song Brocade fabric had the EMI shielding effectiveness value around 50 dB, which was supported by the low surface resistance less than 40 Ω. The excellent NFC shielding of the Ag-PA yarn-incorporated Song Brocade was also found. The ultraviolet protection factor (UPF) value of the Ag-PA yarn-incorporated Song Brocade fabric was higher than 190. The air permeability and the evaporation rate of the Ag-PA yarn-incorporated Song Brocade fabric was higher than 99 mm/s, and 1.4 g/h, respectively. As a result, the Ag-PA yarn-incorporated Song Brocade fabrics were proposed for both the personal and the industrial scale utilization.

## 1. Introduction

With the rapid development of the electronic communication technology, the electronic devices became common in the daily life. Along with the development of the electronic communication, the electromagnetic (EM) radiation generated by the ubiquitous electronic devices also has a negative effect on the human life [1]. The long-term exposure to EM radiation could endanger people’s health [2,3]. It was revealed that the various slight or serious negative effects on the human body have been investigated when someone has been near the mobile base station for a certain time. Additionally, the personal information stored, e.g., in credit cards and other electronic cards may be stolen by using near field communication (NFC) technology, which is based on the EM radiation [4,5,6,7]. To protect electrical equipment and human body from these damages, the fabrics with enhanced electromagnetic interference (EMI) have provided a solution. These fabrics were usually realized by incorporating the metal materials into the fabrics [8,9]. Shielding of EM waves was here achieved by the absorption and reflection of EM radiation in the metal-incorporated fabrics [10,11].

Various methods have been used for the preparation of metal-incorporated fabrics to enhance the EM shielding effectiveness (*SE*), including the coating method (dip coating, the sputtering coating, the electroless plating…) and the weaving technology by using conductive yarns [12,13,14,15,16]. Both the dip coating and the sputtering coating were convenient to prepare the EMI fabrics. The electroless plating technology was realized based on the autocatalytic deposition and simultaneous reduction where the metal ions in the bath were reduced with the catalyst and was suitable for the preparation of the ultralight EMI fabric [17,18,19]. However, the coating technology altered surface chemistry and permeability of the fabric [20]. The friction loss was also the factor affecting the lifetime coated fabric for EMI, which strongly depended on the bonding between the metal materials and the fibers. Furthermore, the surficial patterns of the metal-incorporated fabrics with EMI realized via the surface coating methods was significantly altered when compared with the uncoated fabrics, which strongly limited such metal-incorporated fabrics for the industry rather than the daily use. Besides, the moisture content in the room condition was also a factor to affect the stability of EMI fabric [21]. Opposing to the various coating methods, the weaving method by using conductive yarns to prepare the EMI fabrics could provide a series solution for the problems. However, various research work focused on the effect of the porosity, thickness, yarns type and layers of the EMI fabrics on the final EM *SE* [9,22,23,24,25,26,27]. It was also concluded that the fabrics with conductive yarns were woven with basic structure, like plain, twill, honeycomb, end satin etc. Since the fabric structure was simple, the visible surficial patterns of the EMI fabrics were limited, which had less attraction for the customers. To date, there have been a few reports related to the complicated patterns on the metal-incorporated fabrics. 

The Song Brocade fabric was based on the unique structure, which arose from the Song Dynasty of China [28,29]. The main materials for the Song Brocade fabric were the mulberry silk [30]. The Song Brocade fabric was mainly fabricated based on the two traditional types of weaving [29]. The weave of Song Brocade was weft backed, from two groups of warp and multiple groups of wefts. In details, one group was called the ground warp, which was made of refined and dyed mulberry silk, and the other group was called the face warp, which was usually made of a fine single raw silk. The ratio of the ground warp and face warp was mostly 3, and sometimes the ratio could be 2, 4, 6 etc. [31]. Compared with the basic fabric structure, various patterns of the Song Brocade fabrics could be prepared by adjusting the number of the weaving cycles, including the pattern of ‘key brick’, ‘swastika’, and ‘shou’ etc. From this point of view, the weaving of the Song Brocade fabric by using the metal-incorporated yarns was the prospective alternative for the EMI fabrics. 

In this work, two Song Brocade fabrics were successfully prepared. One was the Song fabrics woven by using the polyester (PET) yarns and the silk yarns, and the other one was woven by using the silver-plated polyamide (Ag-PA) yarns and the silk yarns. Two fabrics were fabricated with the same weaving structure where the ‘swastika’ (卐) appeared as the surficial pattern. The structure similarity of the surficial patterns of both samples were investigated via Python software [32]. The EMI property and the ultraviolet (UV) shielding of both samples were evaluated. The surface resistance of both samples was also measured. Besides, the air permeability and the textile moisture evaporation rate of samples were measured.

## 2. Experimental

### 2.1. Materials 

Three yarns were purchased from Suzhou Shangjiukai Company (Suzhou, China), including the raw silk, the cooked mulberry silk, and the polyester (PET) yarns. The silver-plated nylon 6 (Ag-PA) yarns were purchased from Suzhou Shangjiukai silk technology culture co., ltd. (Suzhou, China). To ensure the visibility of the designed pattern on the surface of the Song brocade fabric, the yarns were proposed to have different colors. The details of the yarns were shown in Table 1. 

### 2.2. Fabrication of Song Brocade Fabric

**Figure 1:**
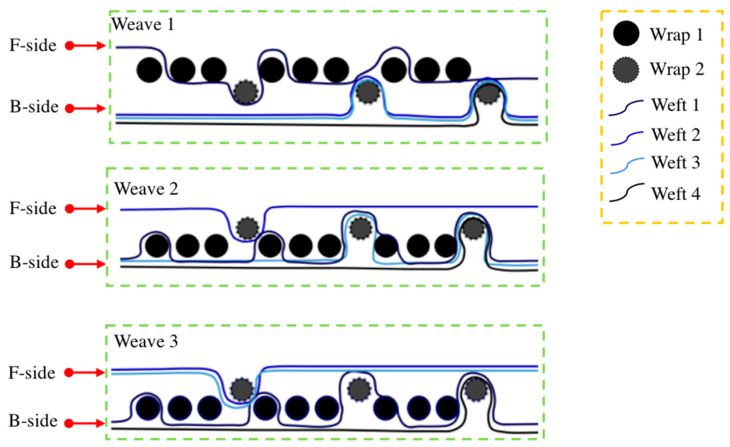
Fabric weaves and structure of the Song Brocade fabric (schematic cross-section).

To fabricate the Song Brocade fabric, it was necessary to design the fabric structure in the weaving system. As shown in Figure 1, the three kinds of weaves were involved in the weaving system, which consisted of two groups of warps and four groups of wefts. The ratio of warp 1 to wrap 2 was 3 and the ratio of four groups of wefts was 1. In detail, the structure of the Song Brocade fabrics was described as followings:

(1) Weave 1 showed the ground pattern. The surface layer consisted of warp 1 and weft 1 in 2/1 twill, the surface of the fabric showed the mixed color of warp 1 and weft 1. The middle layer consisted of warp 2 and weft 2 and weft 3 in 1/2 twill, and the inner layer consists of warp 2 and weft 4 in 2/1 twill.
(2) Weave 2 was aimed to present the ‘卍’, which was a classical Chinese pattern. The surface layer consisted of warp 2 and weft 2 in 1/2 twill, and the surface of the fabric showed the mixed color of warp 2 and weft 2. The middle layer consisted of warp 1 and weft 1 and weft 3 in 1/2 twill, and the inner layer consisted of warp 1 and weft 4 in 2/1 twill.
(3) Weave 3 showed the cross-hatching pattern. The surface layer consisted of warp 2 and weft 2 and weft 3 in 1/2 twill, and the surface of the fabric showed the mixed color of warp 2 and weft 2 and weft 3. The middle layer consisted of warp 1 and weft 1 and weft 1 in 1/2 twill, and the inner layer consists of warp 1 and weft 4 in 2/1 twill.

**Figure 2:**
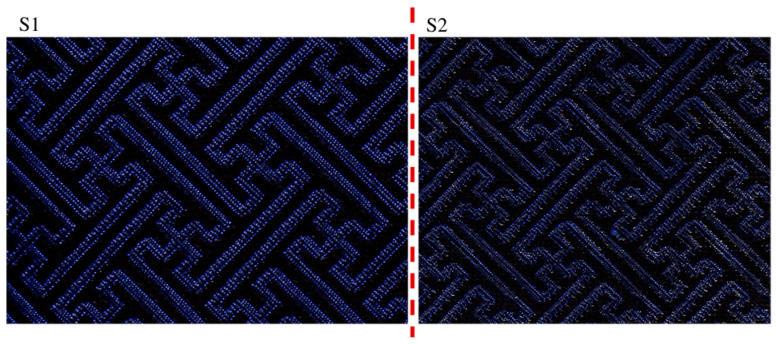
The optical images of the prepared sample S1 and S2 under standard light source.

Table 2 gave the details of the yarns in the prepared Song Brocade fabrics. By using the textile CAD technology, two Song Brocade fabrics were successfully fabricated by jacquard loom. The details of the two Song Brocade fabrics were shown in Table 3. Since the Song Brocade fabric had the different surficial patterns on both sides, the fabric side with surficial pattern ‘卍’ was set as front side (F-side) and the opposite side was set as back side (B-side). It was found that the sample S2 had the higher thickness value than the sample S1. The main reason could be that the Ag-PA yarns of the sample S2 were thicker than the PET yarns of the sample S1. Under the same light source, the physical images of the two samples were shown in Figure 2. It was found that there was no obvious difference in the surficial pattern and the color between two samples. 

### 2.3. Characterization

#### 2.3.1. Analysis of the Morphology of the Song Brocade Fabric

The morphology of the Song Brocade fabric was characterized by using scanning electronic microscopy (SEM) (VEGA TESCAN Inc., Licoln, NE, USA) with the voltage of 20 kV and the laser optical microscopy (LOM) (OLS5000 LEXT, Tokyo, Japan). Since it was not the single layer of the two designed Song Brocade fabrics, the surficial structure was the objective which appeared in the expected ‘卍’ patterns, the structural similarity index measurement (SSIM) of the designed surficial patterns (Figure 2) was calculated via Python software (Version 3.9.6, Beaverton, OR, USA) [23]. By transforming the photos into vectors, the comparison was carried out and the cosine distance (ranged from 0 to 1) based on the mean values, variances and covariance between arrays of image pixels was obtained. If the cosine distance was close to 1, the higher structural similarity between the two surficial patterns was found. Besides, the energy-dispersive X-ray spectroscopy (EDS) was also used to characterize the Ag-PA distribution in the sample S2. 

#### 2.3.2. Evaluation of Electromagnetic Shielding Effectiveness (EM SE)

**Figure 3:**
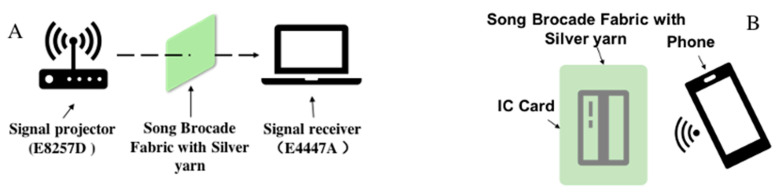
Evaluation of NFC shielding for the prepared samples ((**A**): The standard measurement and (**B**): the measurement with mobile phone and IC card).

The EM *SE* of the fabricated samples S1 and S2 were evaluated by using the E8257D signal generator (Keysight Technologies, Santa Rosa, CA, USA) and the E4447A spectrum analyzer (Keysight Technologies, Santa Rosa, CA, USA), which followed the standard SJ20524-1995 [33]. The device was schemed in Figure 3A. The electromagnetic waves in this measurement ranged from 30 MHz to 3000 MHz. Since the F-side and the B-side of both the sample S1 and the sample S2 were different in their inherent materials and fabric structures, the F-side and the B-side of both the sample S1 and the sample S2 were measured. The measurement was performed under an external environmental condition with the room temperature of 23 ± 2 °C and the humidity of 65 ± 5%. As a result, the EM *SE* is given by the Equation (1) as a logarithmic ratio between the plane field intensity of radiated wave *P*_1_ and plane field intensity of transmitted wave *P*_2_. The *SE* shielding percentage (*SE*%) was also evaluated by using the Equation (2):

(1) $$SE=10\;\log(P_{1}/P_{2})$$

(2) $$SE\%=\left(1-10^{\frac{-SE}{10}}\right)\times 100\%$$

In addition, an experiment was designed to evaluate near field communication (NFC) shielding of the samples by the built-in NFC data reading function in the mobile phone and the integrated circuit (IC) card. The details were shown in Figure 3B. By wrapping the IC card by the sample S1, and S2, respectively, the signal reception of the built-in data reading function in the mobile phone away from the IC card with distance ranging from 0–40 cm was recorded. 

#### 2.3.3. Evaluation of Electrical Conductivity 

It was noticed that there were various methods to evaluate the electrical conductivity. The two probes method was considered as the most common way to measuring the resistance of the conductive materials [34]. In this case, the surface conductivity (Ω) of the Song Brocade fabric was measured according to the two probes method by using the RMS mini multimeter (EXTECH, Waltham, MA, USA).

#### 2.3.4. Evaluation of Ultraviolet (UV) Shielding 

The UV shielding of the prepared samples was evaluated by using the YG(B)912E textile ultraviolet tester (Wenzhou, China), according to standard GB/T 18830-2009 [35]. The measurement was performed under an external environmental condition with the temperature of 20 ± 2 °C and the humidity of 65 ± 5%. The wavelength ranged from 280 nm to 400 nm was used and the transmission with intervals of 5 nm was recorded. The ultraviolet protection factor (UPF) values of the samples were calculated by using the built-in software [36]. Each sample was measured for 5 times for the statistical analysis.

#### 2.3.5. Evaluation of Air Permeability 

The air permeability of the prepared samples was evaluated by using YG(B)461E digital fabric permeability tester (Wenzhou, China), according to standard GB/T 5453-1997 [37]. The measurement was performed under an external environmental condition with the temperature of 20 ± 2 °C and the humidity of 65 ± 5%. Each sample was measured for 5 times for the statistical analysis. 

#### 2.3.6. Evaluation of Moisture Evaporation Rate 

The moisture evaporation rate of the samples was evaluated by using DR290G textile moisture evaporation rate tester (Wenzhou, China), according to standard GB/T 21655.1-2008 [38]. The measurement was performed under an external environmental condition with the temperature 20 ± 2 °C and the humidity of 65 ± 5%. The samples were placed in the sink with water for 10 min. Then, the sample was taken out and dried naturally. The mass of the wetted sample was recorded each 5 min until the change of the mass was not more than 1% for two consecutive weighing. Each sample was measured for 5 times for the statistical analysis. 

## 3. Results and Discussion 

### 3.1. Morphology of the Designed Pattern 

**Figure 4:**
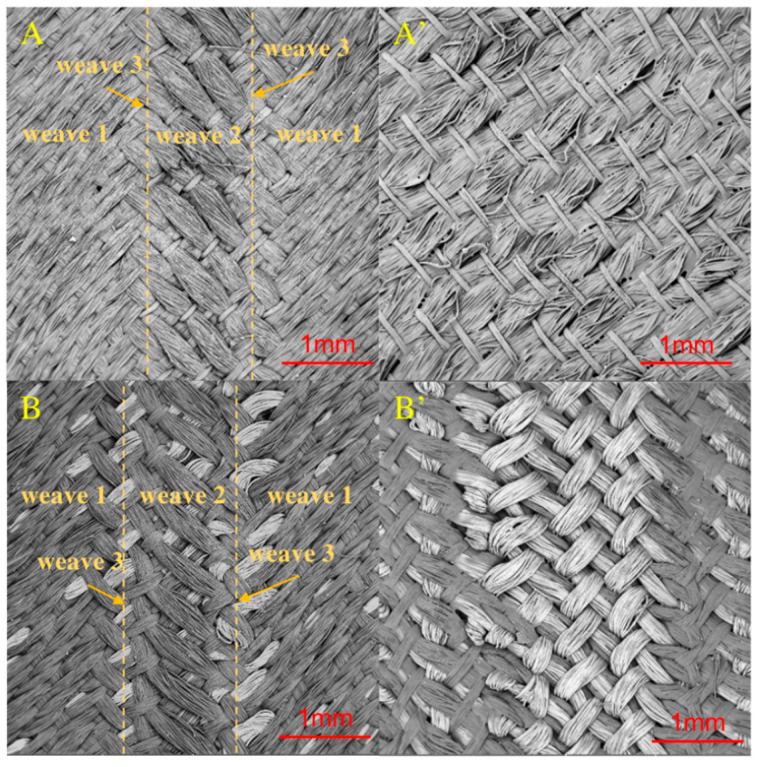
Morphology of the two Song Brocade fabrics from SEM ((**A**,**A’**): the front side and back side of sample S1; (**B**,**B’**): the front side and back side of sample S2).

**Figure 5:**
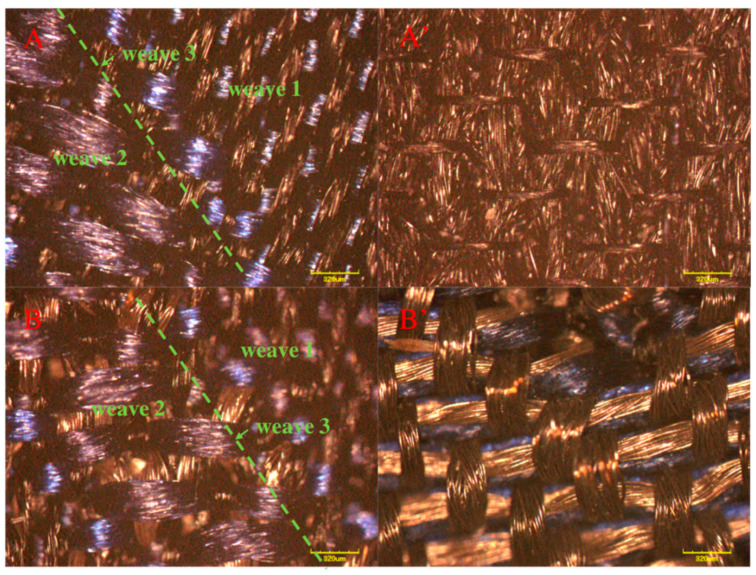
Morphology of the two Song Brocade fabrics from LSM ((**A**,**A’**): the front side and back side of sample S1; (**B**,**B’**): the front side and back side of sample S2).

Figure 4 and Figure 5 presented the morphologies of the sample S1 and sample S2. The different weaving structures (weave 1 and weave 2) were obviously observed. Since the weave 3 was set as the cross-hatching pattern, it appeared as a line. In detail, all the weaving structures of both sides in the designed two Song Brocade fabrics were observed as same, which corresponded to Figure 1. Additionally, there was a visible difference in the comparison between the back sides of the two designed Song Brocade fabrics. The main reason was caused by the different fineness values of the yarns. The calculated SSIM value for the comparison between the sample S1 and the sample S2 was calculated as 0.987, which supported the patterns of the two fabrics were similar. From this point, both the weaving structure and the surficial patterns were not significantly affected by the yarn types in the Song Brocade fabric structure. 

**Figure 6:**
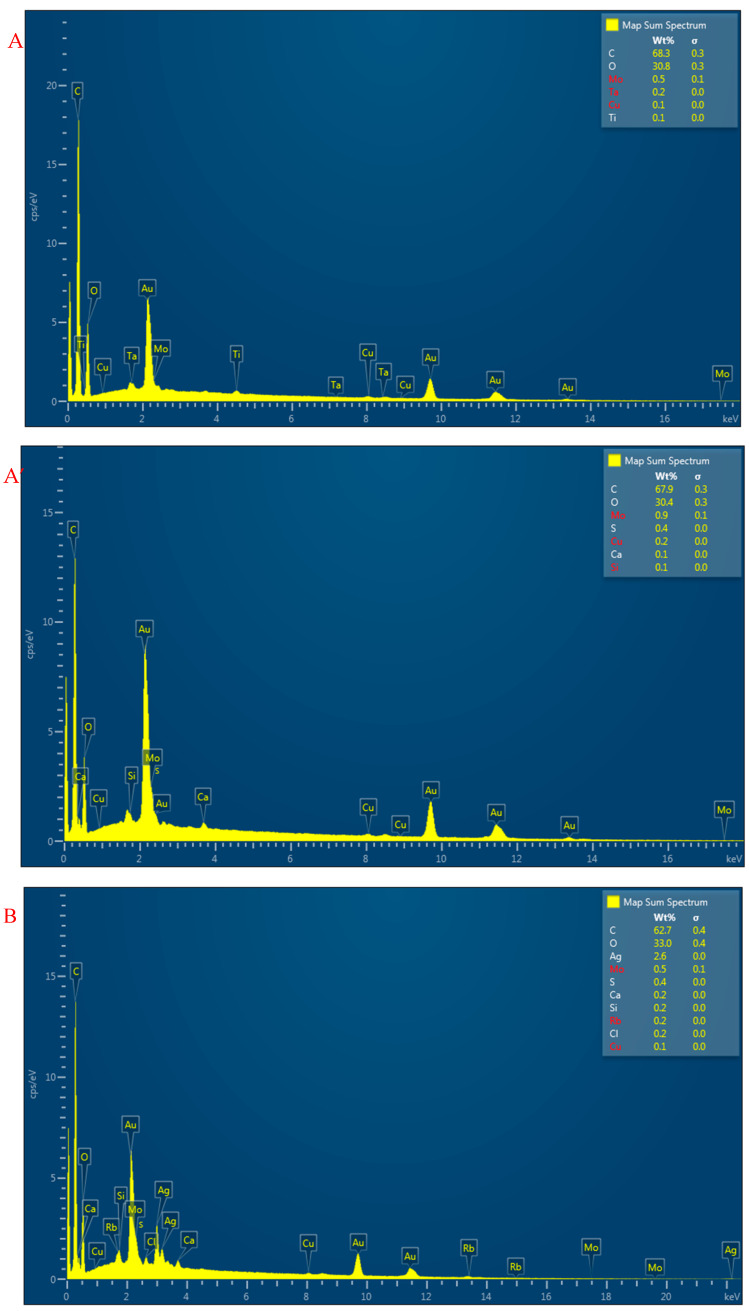

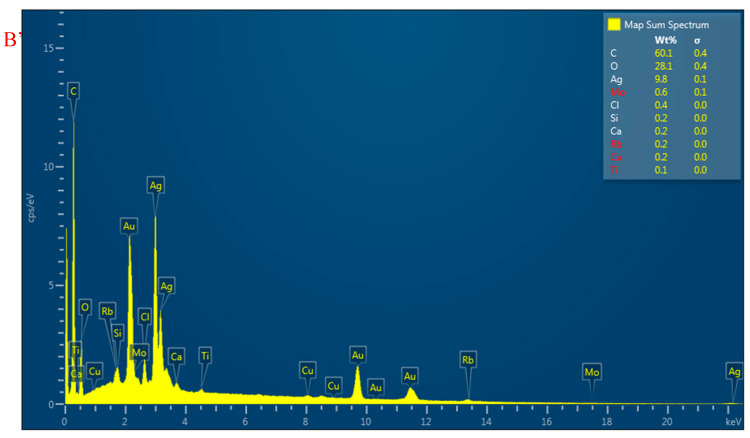
SEM-EDX results for both sides of two Song Brocade fabrics ((**A**,**A’**): the front side and back side of sample S1; (**B**,**B’**): the front side and back side of sample S2).

Figure 6 provided the elements of the sample S1 and the sample S2. Obviously, only the sample S2 had the Ag components. Besides, it was found that the back side of the sample S2 had the Ag content of 9.8 wt%, which was higher than the sample S1 with the Ag content of 2.6 wt%, which was consistent with the Song Brocade fabric structure. More Ag-PA yarns were distributed in the back side of the sample S2. Besides, there were other components (e.g., Mo, S, Si, Ca, Rb, Cu, Ti…) to be detected, which was contributed from the dyes and pigments on the yarns. 

### 3.2. Analysis of EMI Shielding

**Figure 7:**
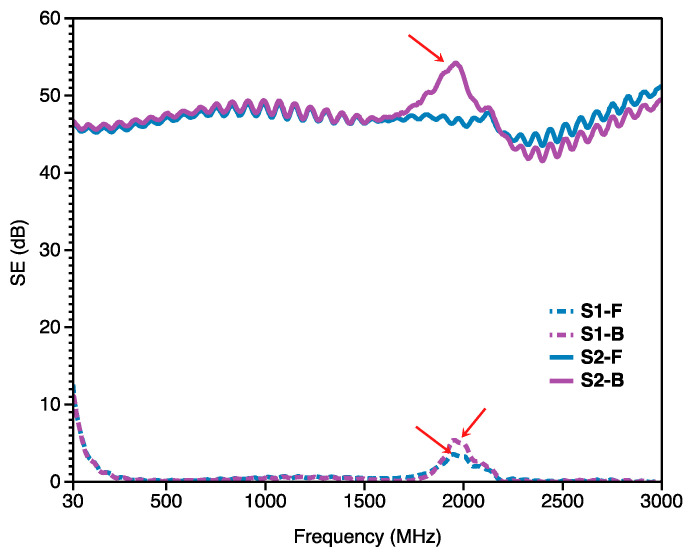
EM *SE* values versus frequency of EM curves for the Song Brocade Fabrics.

Figure 7 presented the EM *SE* values with the frequency values ranging from 30 MHz to 3000 MHz of the two Song Brocade fabrics. It was found that the sample S1 has very small the EM *SE* values (<10 dB) over the whole frequency, while the sample S2 has the EM *SE* value ranging from 40 dB to 55 dB which was comparable with some published research works related to EMI fabric [22,23,24,25,26]. In detail, there was an obvious peak over the whole frequency for both sides of the sample S1. The main reason may be the Song fabric structure and the inherent property dyes of the different yarns. For the sample S2, there was also an obvious peak for the back side of the sample S2 (S2-B) while there was no peak at the same frequency for the front side of the sample S2 (S2-F). The main reason could be caused by the more content of the Ag-PA yarns in the back side than the front side of the sample S2. Especially, the calculated EM *SE* values and the *SE*% values of the sample S2 at the selected frequency were shown in Table 4. It was found that the *SE*% values of the sample S2 were 99.99% at the selected frequency. According to the common classification, the sample S2 (the Ag-PA yarn-incorporated Song Brocade fabric) was evaluated in the ‘excellent’ category for both general and professional use. 

Since the Ag-PA yarn-incorporated Song Brocade fabric (sample S2) had the small thickness of 0.542 mm, the other parameters including the EM *SE* value normalized to the thickness (*SE/t*), the specific shielding effectiveness (*SSE*) and the absolute shielding effectiveness (*SSE/t*) were evaluated. In detail, the *SE/t* (dB cm^−1^), *SSE* (dB cm^3^ g^−1^) and *SSE/t* (dB cm^2^ g^−1^) were calculated according to Equations (3)–(5) and given in Table 4, where the *h* was the thickness and *S* was the areal density in Table 3. It was found that both *SSE* value and *SSE/t* value of the sample S2 at specific frequency was much smaller by comparing with other EMI fabrics [39,40], while the *SE/t* value was stable and higher than 960 dB cm^−1^. The difference was caused by the combination of the Song Brocade fabric components and the Song Brocade fabric structure. There were three type yarns including the conductive Ag-PA yarns and two nonconductive silk yarns in the sample S2. As a result, *SSE* value and *SSE/t* value were significantly reduced. However, the Ag-PA yarns were orderly woven in the fabric structure and could be observed along the thickness direction, which contributed to the higher *SE/t* value:

(3) SE/t=EM SE/h

(4) SSE=EM SE/(h·S)

(5) SSE/t=EM SE/S

**Figure 8:**
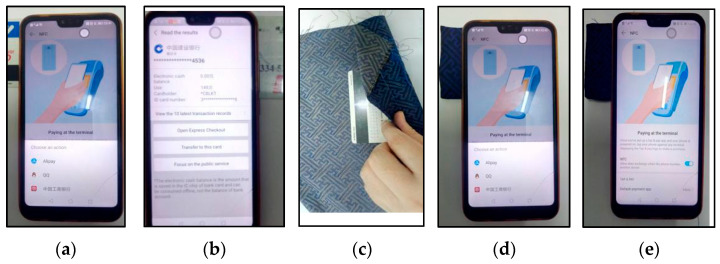
Practical measurement with NFC function in mobile phone ((**a**): the mobile phone read unprotected IC cards, (**b**): the details of IC card, (**c**): wrapping IC cards with the sample S2, (**d**): the reading situation of the mobile phone from the wrapped IC card and (**e**): no signal response shown on the mobile phone).

Additionally, the measurement of the shielding of the NFC data reading by using the mobile and the IC card were carried out to reveal the good compatibility of the prepared silver yarn-incorporated Song Brocade fabrics with various products. Figure 8 presented the different situations of the data reading of the phone from the IC card and more details were given in the video document (Supplementary Materials). As a result, the IC card was covered by the sample S2 and there was no signal shown in the mobile phone, which corresponded to the EM *SE* analysis. 

### 3.3. Evaluation of the Conductivity 

By using the multimeter device, it was found that there was no value reading for the sample S1, while the values were found to range from 1 to 40 Ω for the sample S2. The results were consistent with the EMI results. 

**Figure 9:**
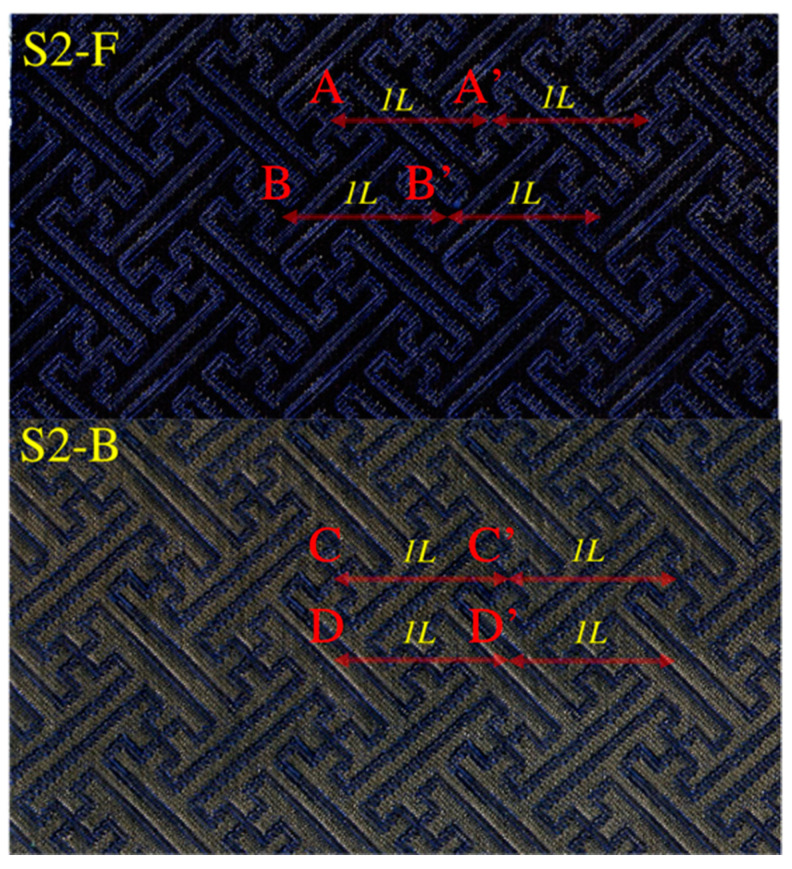
Description for the measurement of the surface resistance of the sample S2.

**Figure 10:**
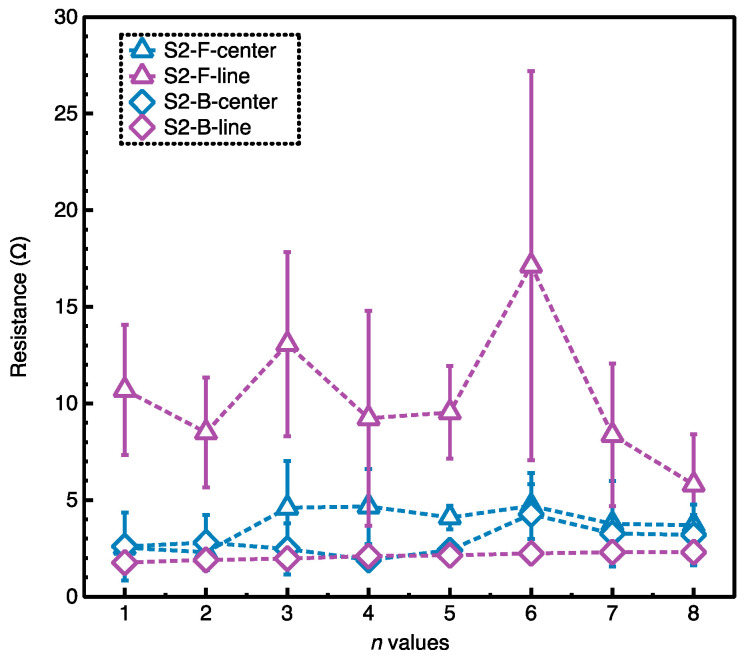
The change of resistance with distances for the silver-incorporated Song Brocade fabric (the sample S2).

Additionally, the S2 had the complicated structure, as described in the analysis. To reveal the electrical resistance stability of the sample S2, the measurement with different distances between the measured points on both sides of the sample S2 was carried out. Since the sample was based on the ordered weaving structures (weave 1, weave 2 and weave 3), the distance between the measured points was set according to the fabric weaves. Besides, it was noticed that the pattern ‘卐’ was based on the weave 1 and the area around the pattern ‘卐’ was based on the weave 2. Therefore, two measurements were carried out based on the weave 1, and weave 2, respectively. In detail, the center of the close pattern ‘卐’ on both sides was chosen as one measured points (A and A’, and C and C’ in Figure 9), and the straight line around the pattern ‘卐’ on both sides was chosen as the other measured points (B and B’, and D and D’ in Figure 9). The distance between the two close measured points was considered as *L* (2.5 cm). Then, the change of the distance between the two measured points was set as *nL* (*n* was the integer). The results of the resistance with different distances between the measured points were given in Figure 10. It was found that the maximum surface resistance of the sample S2 was less than 30 Ω, which contributed to the excellent EM *SE.* Besides, the distance between the measured points had little effect on the surface resistance, which supported the highly stable electrical conductivity. In addition, the surface resistance of the F-side was slightly higher than the surface resistance of the B-side. The reason was that the B-side of the sample S2 had more Ag content than the front side according to the designed fabric structure. 

### 3.4. Evaluation of UV Shielding Property

**Figure 11:**
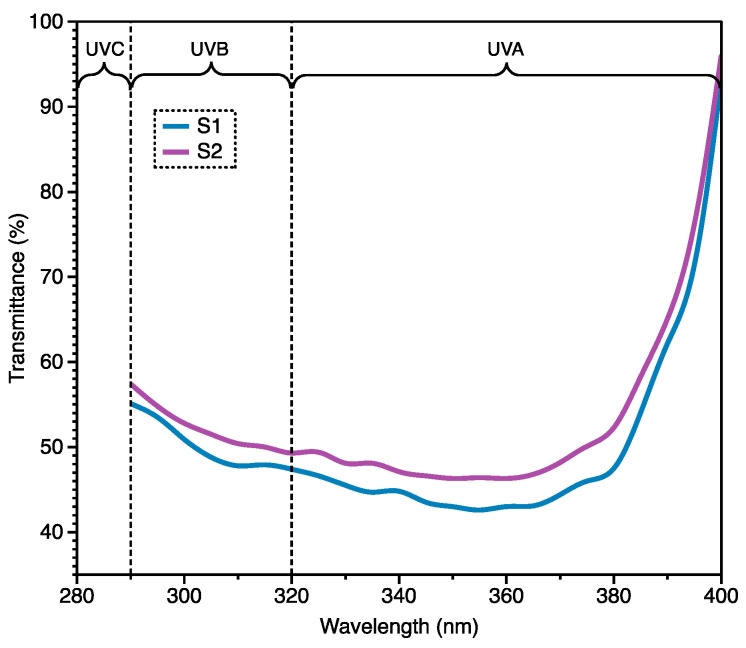
The UV transmittance of the Song Brocade fabrics with and without Ag-PA yarns over 290–400 nm.

The UV measurement of both sample S1 and S2 were shown in Figure 11, including the ultraviolet radiation A (UVA), the ultraviolet radiation B (UVB) and the ultraviolet radiation C (UVC). The sample S1 and S2 shared the similar plot of the transmittance against wavelength. In details, the UVA and UVB transmission and the UPF values of both sample S1 and S2 were shown in Table 5 The sample S1 had the UPF value of 206.64 and the sample S2 had the UPF value of 196.22. Usually if the UPF value of the fabric was equal to or higher than 50, the block from the 98% UV radiations was realized. Therefore, both S1 and S2 had the excellent UV shielding from this point. 

It was interesting that the sample S1 without Ag-PA yarns already had the higher transmittance in both UVA and UVB and its UPF value was also higher when compared with sample S2. For the UV shielding effectiveness, the inherent property of material and the fabric structure had the significant effect. In this case, the UV shielding effectiveness of the sample S1 was attributed to the high areal density. By comparing with the S1, the sample S2 had the lower areal density although there were Ag-PA yarns inside. Then, it was hard to determine the contribution from the Ag-PA yarns to the UV shielding effectiveness. 

### 3.5. Air Permeability

The measured air permeability of both sample S1 and S2 were shown in Table 6. It was found that the sample S1 had the air permeability of 34.06 mm/s and the sample S2 had the air permeability of 99.42 mm/s. Therefore, the sample S2 had the better air permeability than the sample S1. 

The main reason could be the difference in the yarn type, yarn density and the weaving structure of both samples, which was described in the Section 2.1 and Section 2.2. The PET yarns for the sample S1 were 300 den and the Ag-PA yarns for the sample S2 were 90 den, while both samples had the same warp density values as well as the weft density values. Then, the porosity of the sample S2 was considered to be slightly higher than the sample S1. As a result, the sample S2 had the higher air permeability. 

### 3.6. Evaluation of Water Evaporation 

**Figure 12:**
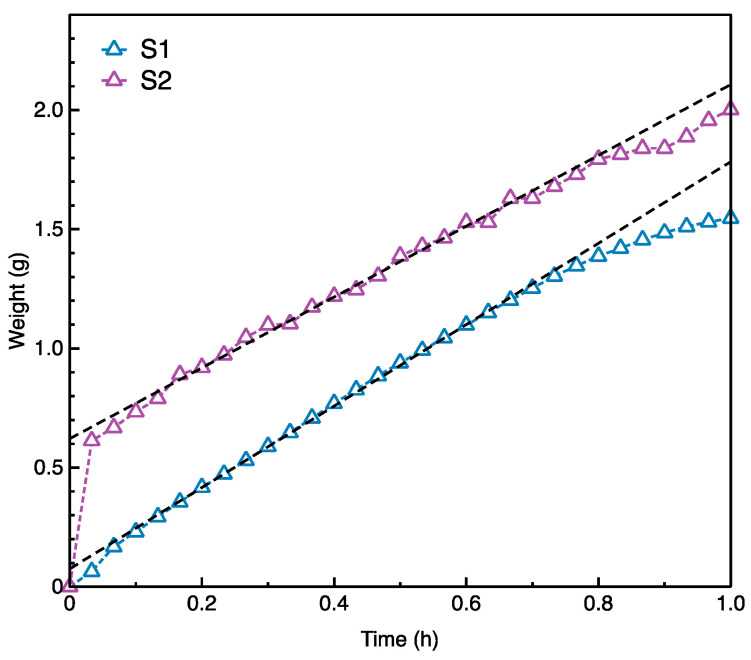
Evaluation of water evaporation rate of the sample S1 and S2.

The water evaporation of both sample S1 and sample S2 in 1 h were recorded and shown in Figure 12. Additionally, two formulas were modeled to characterize the water evaporation according to the standard GB/T 21655.1-2008, which was shown in Table 7. It was found that the sample S1 had the evaporation rate of 1.709 g/h, which was higher than the sample S2 with evaporation rate of 1.486 g/h. 

Similar as in the case of air permeability, the main reason could be the difference in the yarn type, yarn density and the weaving structure of both samples, which was described in the Section 2.1 and Section 2.2. Both samples had the same fabric structure and the sample S1 had the lower porosity than the sample S2. The remaining water in the sample S1 was considered to be less than the sample S2. Besides, the PET yarns were used in the sample S1, while the Ag-PA yarns were used in the sample S2. The interaction between the surface of the PET yarns and the water was much lower than the interaction between the surface of the Ag-PA yarns and the water. Therefore, it was easier for the water evaporation from the sample S1 than from the sample S2. 

## 4. Conclusions

In this work, the Song Brocade fabrics with and without Ag component were successfully fabricated. It was found that the incorporation of the Ag-PA yarns into the Song Brocade fabric did not affect the surficial pattern. It was found that the surface resistance of the Ag-PA yarn-incorporated Song Brocade fabric less than 40 Ω and was highly stable on both sides, which contributed to the excellent electromagnetic shielding with EM *SE* value higher than 54 dB. Since the Ag-PA yarn-incorporated Song Brocade fabric consisted of the Ag-PA yarns and two silk yarns, the specific shielding effectiveness and absolute shielding effectiveness were much lower. However, the well-distribution of the Ag-PA yarn in the Song Brocade fabric contributed to the high *SE/t* value. The practical test that the signal reading from the IC card covered by the Ag-PA yarn-incorporated Song Brocade fabric to the phone was totally failed, which corresponds to the results of the EMI analysis. The UPF values of the prepared two Song Brocade fabrics were higher than 195, which supported the excellent UV shielding effectiveness. Additionally, both the air permeability value and the water evaporation rate value of the Song Brocade fabric without Ag component was lower. The main reason was caused by the different yarn types in two fabrics. 

**Figure 13:**
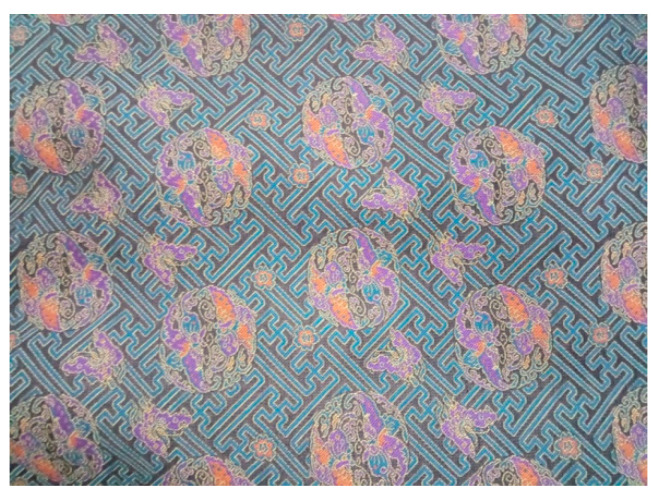
The other Song Brocade fabric with Ag-PA yarns.

Except for the sample with ‘卐’ as surficial pattern shown in this work, the other Song Brocade fabric type with Ag-PA yarns was shown in Figure 13. The surficial pattern was more complicated, which consisted of the ‘flower’ shape and ‘卐’ shape. We proposed that the functional Song Brocade fabric could be applied in the personal protection and the industrial applications. 

## Figures and Tables

**Table 1:** Details of the used yarns.

Yarn Type	Fineness (Denier)	Color
Raw silk	1/20/22 den	Black
Cooked mulberry silk	1/20/22 den	Black
	5/20/22 den	Blue 1, Blue 2, Blue 3
PET	300 den	Brown
Ag-PA	90 den	Black

**Table 2:** Details of the yarns for Song Brocade fabric.

Yarn Code	Yarn Type in the Sample S1	Yarn Type in the Sample S2
Wrap 1	Raw silk (1/20/22 den)	Raw silk (1/20/22 den)
Wrap 2	Cooked mulberry silk (1/20/22 den)	Ag-PA yarn (90 den)
Weft 1	Cooked mulberry silk (5/20/22 den)	Cooked mulberry silk (5/20/22 den)
Weft 2	Cooked mulberry silk (5/20/22 den)	Cooked mulberry silk (5/20/22 den)
Weft 3	Cooked mulberry silk (5/20/22 den)	Cooked mulberry silk (5/20/22 den)
Weft 4	PET Yarn (300 den)	Ag-PA yarn (90 den)

**Table 3:** Details of the prepared Song Brocade fabric.

Sample Code	Warp Density (Root/cm)	Weft Density (Root/cm)	Areal Density (g/cm^2^)	Thickness (mm)
S1	120	120	239	0.438
S2	120	120	194	0.542

**Table 4:** EM *SE* values of the sample S2 at selected frequencies.

Frequency (MHz)	EM *SE* (dB)	EM *SE/t* (dB cm^−1^)	*SSE* (dB cm^3^ g^−1^)	*SSE/t* (dB cm^2^ g^−1^)	*SE*%
30	54.1	998.155	0.015	0.279	99.99
100	54.6	1007.380	0.015	0.281	99.99
300	54.4	1003.690	0.015	0.280	99.99
1000	54.6	1007.380	0.015	0.281	99.99
3000	52.1	961.255	0.015	0.269	99.99

**Table 5:** Results of UV shielding effectiveness from Figure 11.

Sample Code	UVA Transmission (%)	UVB Transmission (%)	UPF
S1	0.51 ± 0.03	0.51 ± 0.02	206.64 ± 8.86
S2	0.54 ± 0.01	0.53 ± 0.02	196.22 ± 4.75

**Table 6:** Results of air permeability.

Sample Code	Air Permeability (mm/s)
S1	34.06±8.19
S2	99.42±3.69

**Table 7:** Evaporation rate of the sample S1 and S2.

Sample Code	Formula	*R* ^2^	Evaporation Rate (g/h)
S1	y = 0.075 + 1.709x	0.999	1.709
S2	y = 0.621 + 1.486x	0.992	1.486

## Data Availability

Data is contained within the article or Supplementary Material.

## Supplementary Materials

The following are a.vailable online at https://www.mdpi.com/article/10.3390/ma14143779/s1.
