# Supplementary material for: A Silver Yarn-Incorporated Song Brocade Fabric with Enhanced Electromagnetic Shielding
Source: Materials (Basel). 2021 Jul 6;14(14):3779. doi: 10.3390/ma14143779 (PMC8307523; doi:10.3390/ma14143779)
Supplement: Supplementary file 1 [file materials-14-03779-s001.zip › materials-1264669-supplementary.pptx]

## Slide 1
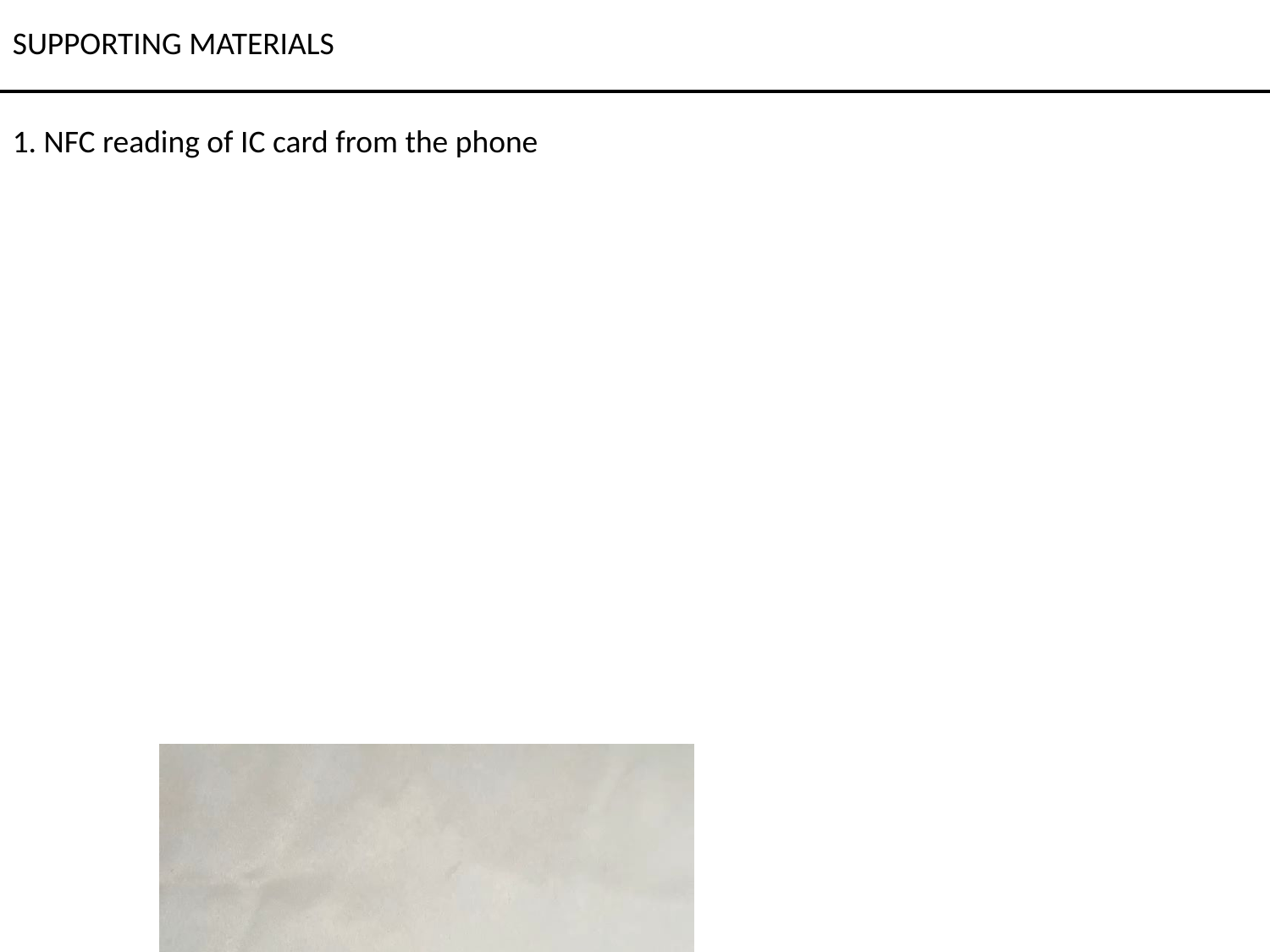

SUPPORTING MATERIALS
1. NFC reading of IC card from the phone

## Slide 2
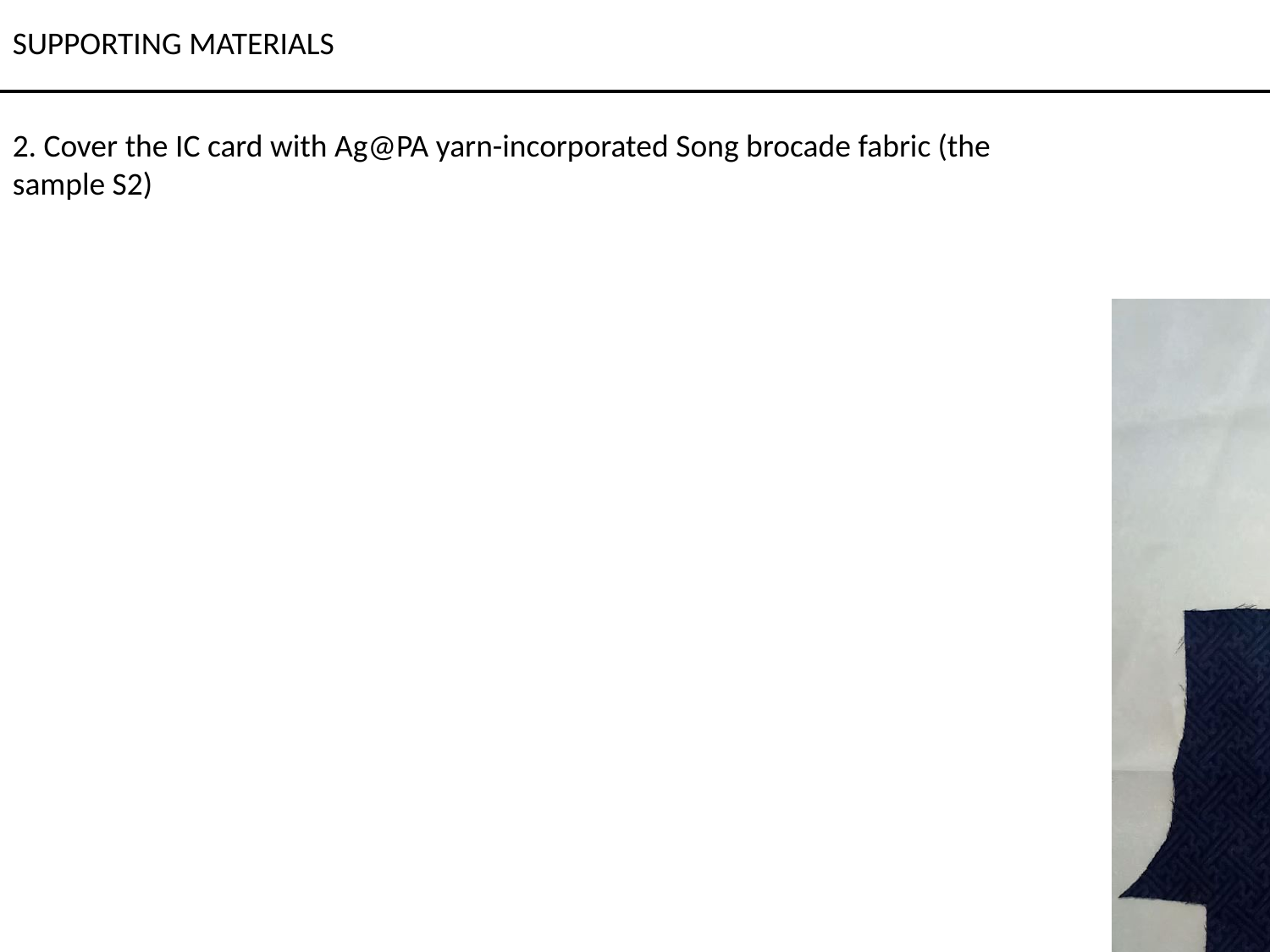

SUPPORTING MATERIALS
2. Cover the IC card with Ag@PA yarn-incorporated Song brocade fabric (the sample S2)

## Slide 3
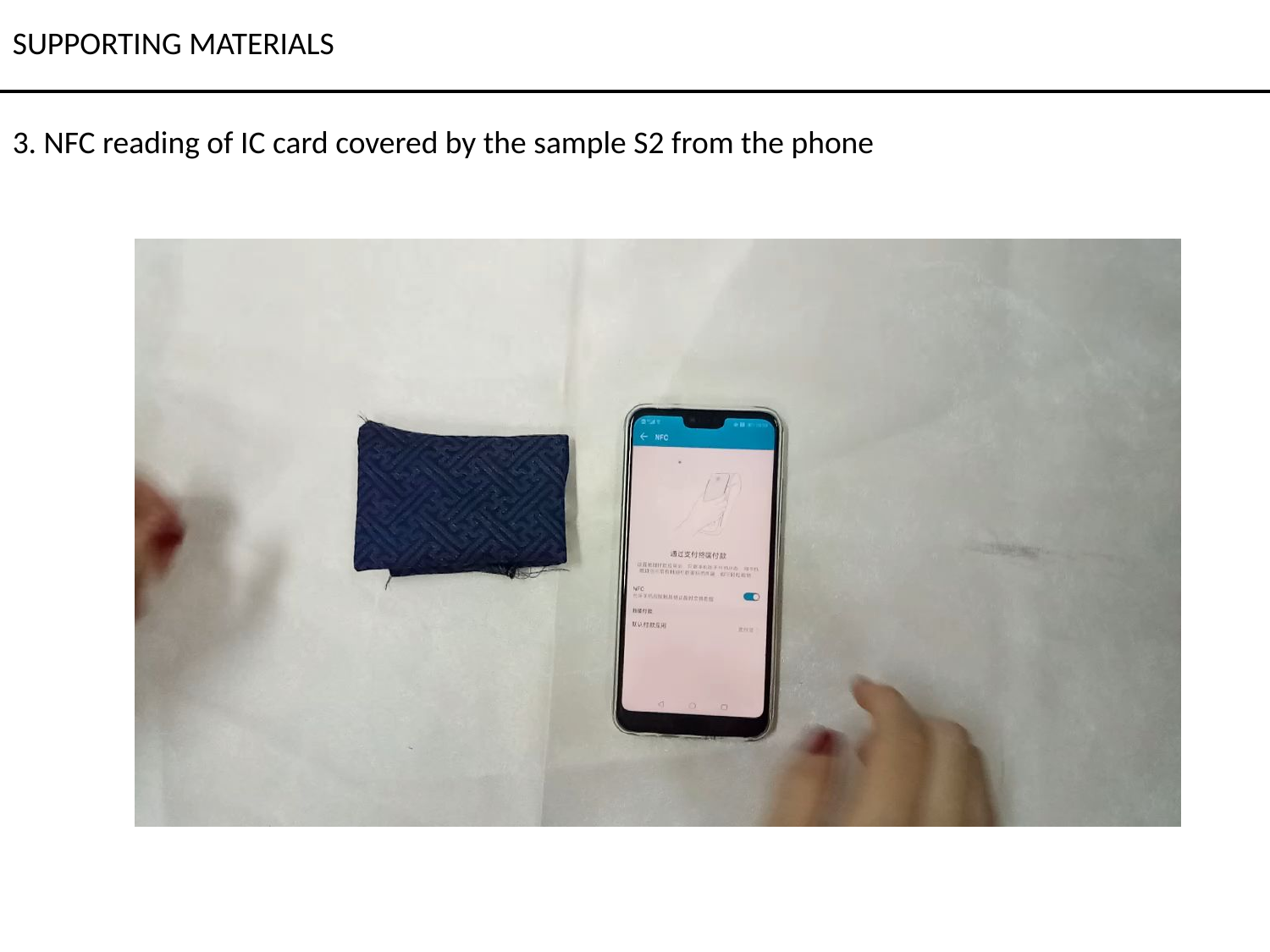

SUPPORTING MATERIALS
3. NFC reading of IC card covered by the sample S2 from the phone
